# The genome sequence of a soldier beetle, *Malthinus flaveolus *(Herbst, 1786)

**DOI:** 10.12688/wellcomeopenres.21086.1

**Published:** 2024-03-01

**Authors:** Mark G. Telfer, Michael F. Geiser, University of Oxford and Wytham Woods Genome Acquisition Lab, Darwin Tree of Life Barcoding collective, Wellcome Sanger Institute Tree of Life Management, Samples and Laboratory team, Wellcome Sanger Institute Scientific Operations: Sequencing Operations, Wellcome Sanger Institute Tree of Life Core Informatics team, Tree of Life Core Informatics collective, Darwin Tree of Life Consortium

**Affiliations:** 1 Independent researcher, Ventnor, Isle of Wight, England, UK; 2 Natural History Museum, London, England, UK

**Keywords:** Malthinus flaveolus, soldier beetle, genome sequence, chromosomal, Coleoptera

## Abstract

We present a genome assembly from an individual female *Malthinus flaveolus* (soldier beetle; Arthropoda; Insecta; Coleoptera; Cantharidae). The genome sequence is 236.7 megabases in span. Most of the assembly is scaffolded into 6 chromosomal pseudomolecules, including the X sex chromosome. The mitochondrial genome has also been assembled and is 19.27 kilobases in length. Gene annotation of this assembly on Ensembl identified 16,617 protein coding genes.

## Species taxonomy

Eukaryota; Opisthokonta; Metazoa; Eumetazoa; Bilateria; Protostomia; Ecdysozoa; Panarthropoda; Arthropoda; Mandibulata; Pancrustacea; Hexapoda; Insecta; Dicondylia; Pterygota; Neoptera; Endopterygota; Coleoptera; Polyphaga; Elateriformia; Elateroidea; Cantharidae; Malthininae; *Malthinus*; *Malthinus flaveolus* Herbst, 1786 (NCBI:txid195224).

## Background

The family Cantharidae, commonly known as “soldier beetles” comprises over 6,000 described species worldwide, of which 41 species are recorded from Britain and Ireland ( Duff, 2018). The British species are grouped into three subfamilies: Cantharidae, with 24 species, Silinae with just one ( *Silis ruficollis*) and the remaining 16 species are classified under Malthininae. The subfamilies of Cantharidae have recently been subject to a molecular phylogenetic study ( Motyka *et al.*, 2023). Malthininae includes the smallest and most difficult-to-identify species, and also some of the least-known British soldier beetles. *Malthinus flaveolus* Herbst, 1786 is the most frequently recorded of the four British species in the genus *Malthinus* Latreille, 1806.

In some of the literature from continental Europe, the species is listed under the name *M. punctatus* Fourcroy, 1785, which, however, is a junior homonym. *M. flaveolus* is accepted as the valid name for the species in most recent taxonomic catalogues (e.g. Constantin, 2014; Fanti, 2014; Kazantsev, 2012; Kazantsev & Brancucci, 2007).

The British members of Malthininae can be identified using Duff (2020) or Dahlgren (1979). They include the smallest members of Cantharidae with a body length between 1.5 and 6 mm. A key feature of the British Malthininae are the somewhat shortened elytra, which leave a part of the hind wings exposed when the beetle is resting. This, however, is not true for all members of this subfamily worldwide. *Malthinus* is distinguished from *Malthodes* by the distinctive head shape, with a rather narrow “neck” and protruding eyes (particularly large in the males), located at the widest part of the head. While all British members of *Malthodes* are black or dark brown when fully coloured (often excluding the apices of the elytra and/or the prothorax), *Malthinus* have at least partially yellow legs, and often a mostly yellowish or pale brownish underside. *Malthinus flaveolus* is distinguished from the other three British *Malthinus* species by the combination of colour (entirely yellow legs and underside, bright yellow elytral apices) and the absence of distinct rows of impressed punctures on the elytra (which are present in *M. seriepunctatus* and *M. balteatus*). The pattern of the pronotum is also a good key character: In *M. frontalis*, the pronotum is entirely black; in *M. seriepunctatus* and *M. balteatus*, it features a central hourglass-shaped black pattern on a yellow or reddish-brown background (sometimes reduced to two disconnected triangles at the base and apex); in *M. flaveolus*, the pronotum is yellow with a black stripe or dot on each side of the middle line, not extending to the margins. In some *M. flaveolus* individuals, these two stripes can be expanded into a single black blotch (leaving the margins and parts of the middle line yellow), in others the black stripes are reduced in size, or even completely disappear, leaving the pronotum completely yellow. At 5–6 mm, *M. flaveolus* is one of the largest members of Malthininae in Britain, but still small compared to most Cantharinae. As in most soldier beetles, females are on average larger than males.

*M. flaveolus* is a species endemic to Europe, widely distributed between northern Spain and Russia west of the Urals, including Britain and Ireland and parts of Fennoscandia ( Kazantsev & Brancucci, 2007). In Britain and Ireland, it is widespread and common in England and Wales, relatively scarce in Scotland and Ireland, and seemingly absent from the Isle of Man and all the islands surrounding Scotland ( Alexander, 2003; NBN Atlas Partnership, 2021).

*M. flaveolus* is a species of woodlands and hedgerows, with adults active mainly in June to July ( Bretzendorfer, 2017). Adult beetles are assumed to be predators hunting smaller arthropods on foliage, even though exact observations on the feeding habits of Malthininae are unfortunately still scarce ( Alexander, 1991; Ramsdale, 2002). They are frequently found resting on the underside of leaves of various trees and shrubs, particularly along the edges of forests, and can be collected using a beating tray or a flight interception trap. Larvae of Malthininae are saproxylic predators, usually found under bark and in dead wood, while larvae of Cantharinae usually inhabit leaf litter ( Alexander, 1991). Their larval biology explains why all British members of Malthininae seem to be associated with woodlands, while some members Cantharinae and Silinae frequent more open areas. It may also explain the clear drop-off in abundance between England and the less forested areas in northern Scotland. The larval morphology of *M. flaveolus* was studied by Fitton (1976).

Here we present a chromosomally complete genome sequence for *Malthinus flaveolus*, derived from a female specimen from Wytham Woods, Oxfordshire. It is hoped that this genome will aid research in the taxonomy and phylogeny of the group, as well as potential applications for environmental DNA and research on the pre-imaginal stages.

## Genome sequence report

**Figure 1.**
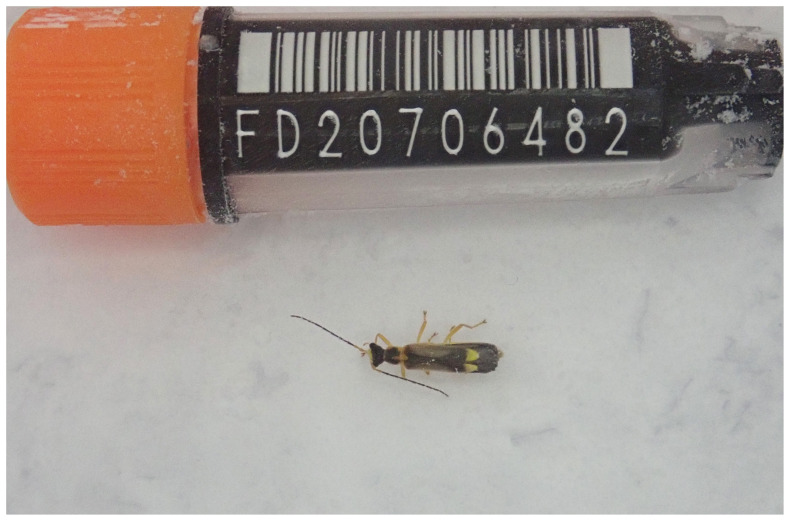
Photograph of the *Malthinus flaveolus* (icMalFlav1) specimen used for genome sequencing.

The genome was sequenced from one female *Malthinus flaveolus* ( Figure 1) collected from Wytham Woods, Oxfordshire, UK (51.77, –1.33). A total of 103-fold coverage in Pacific Biosciences single-molecule HiFi long reads was generated. Primary assembly contigs were scaffolded with chromosome conformation Hi-C data. Manual assembly curation corrected 4 missing joins or mis-joins, reducing the scaffold number by 11.11%.

**Figure 2.**
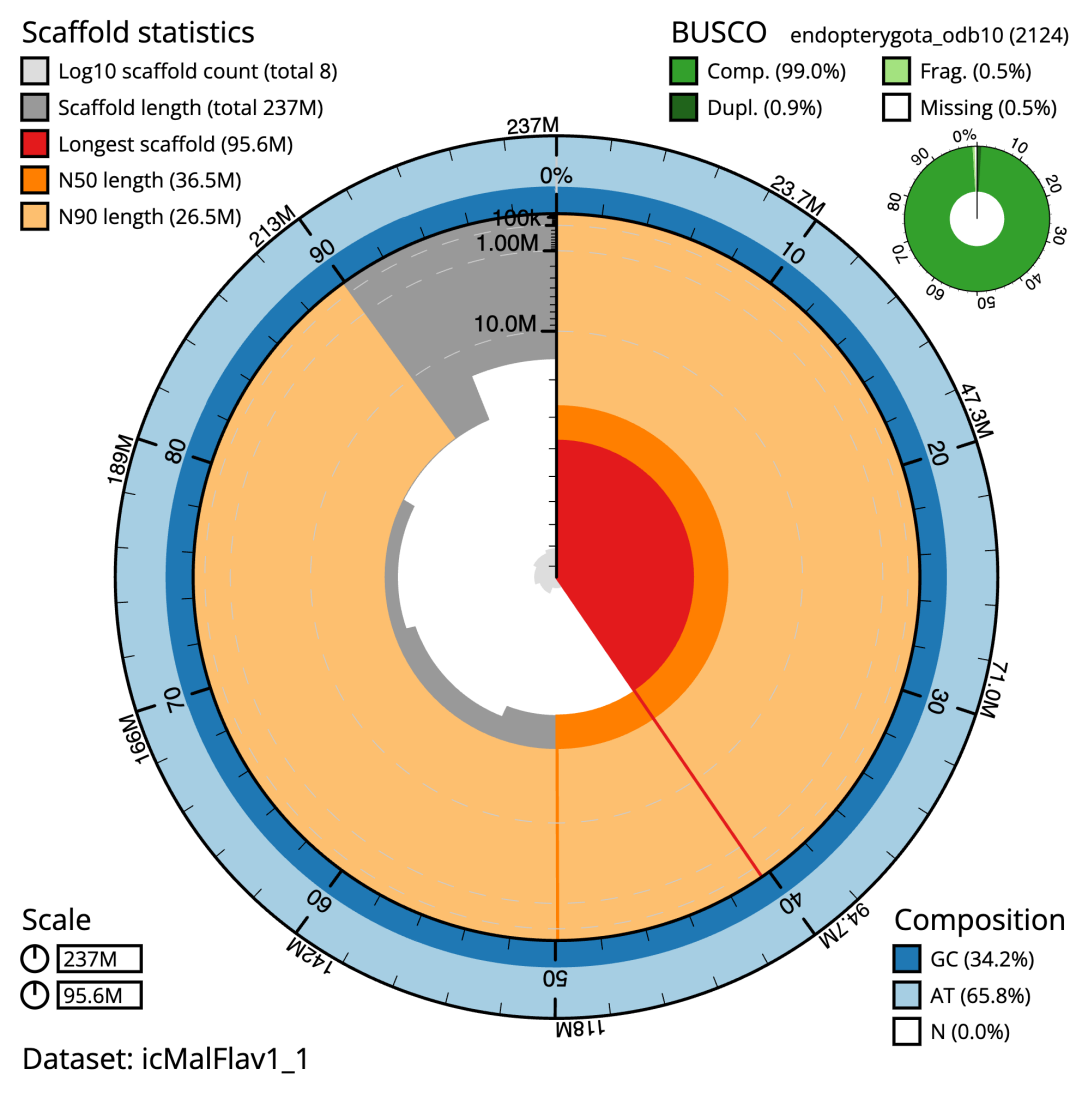
Genome assembly of *Malthinus flaveolus*, icMalFlav1.1: metrics. The BlobToolKit snail plot shows N50 metrics and BUSCO gene completeness. The main plot is divided into 1,000 size-ordered bins around the circumference with each bin representing 0.1% of the 236,723,918 bp assembly. The distribution of scaffold lengths is shown in dark grey with the plot radius scaled to the longest scaffold present in the assembly (95,637,330 bp, shown in red). Orange and pale-orange arcs show the N50 and N90 scaffold lengths (36,462,998 and 26,549,421 bp), respectively. The pale grey spiral shows the cumulative scaffold count on a log scale with white scale lines showing successive orders of magnitude. The blue and pale-blue area around the outside of the plot shows the distribution of GC, AT and N percentages in the same bins as the inner plot. A summary of complete, fragmented, duplicated and missing BUSCO genes in the endopterygota_odb10 set is shown in the top right. An interactive version of this figure is available at https://blobtoolkit.genomehubs.org/view/icMalFlav1_1/dataset/icMalFlav1_1/snail.

**Figure 3.**
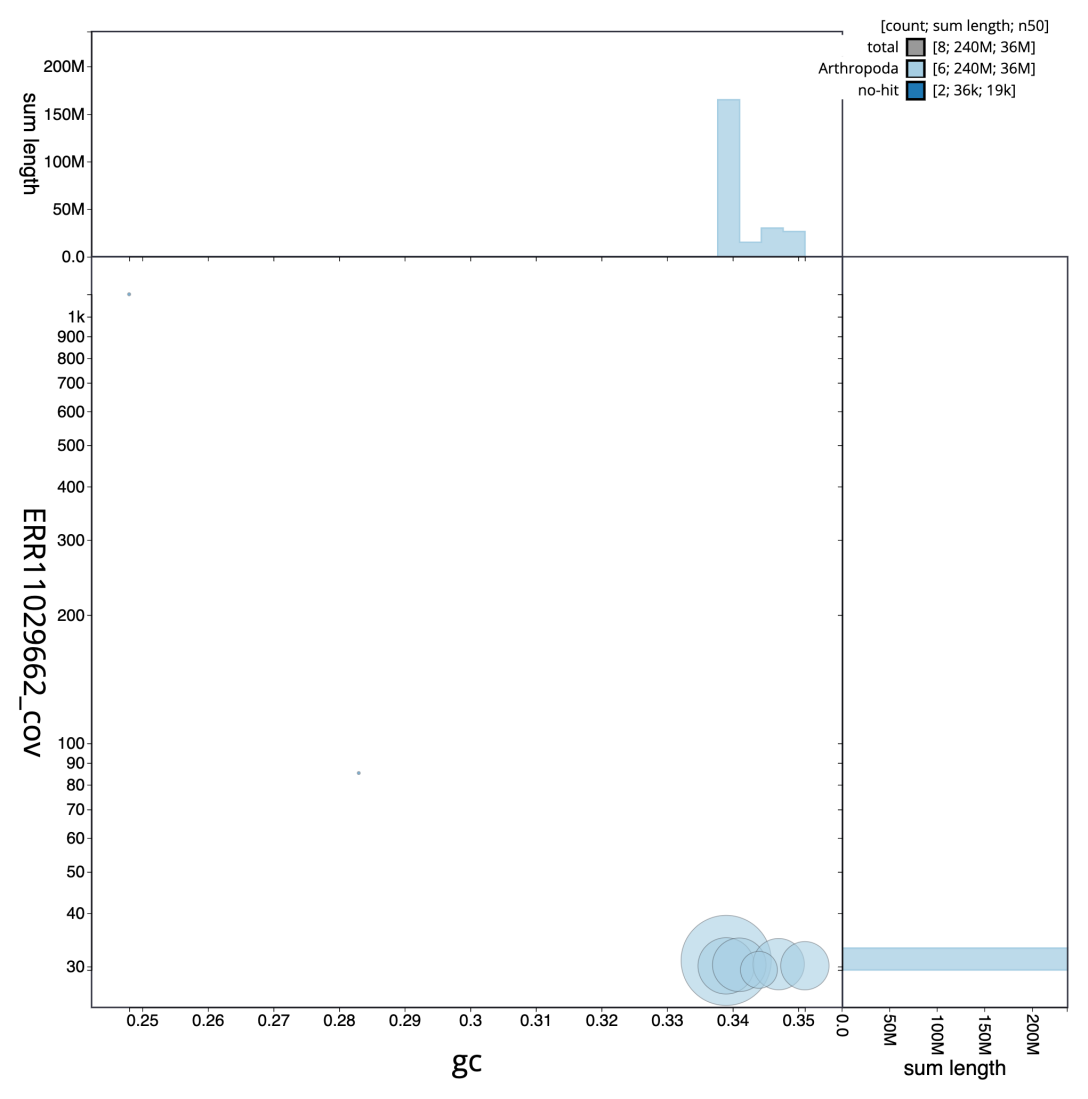
Genome assembly of *Malthinus flaveolus*, icMalFlav1.1: BlobToolKit GC-coverage plot. Sequences are coloured by phylum. Circles are sized in proportion to sequence length. Histograms show the distribution of sequence length sum along each axis. An interactive version of this figure is available at https://blobtoolkit.genomehubs.org/view/icMalFlav1_1/dataset/icMalFlav1_1/blob.

**Figure 4.**
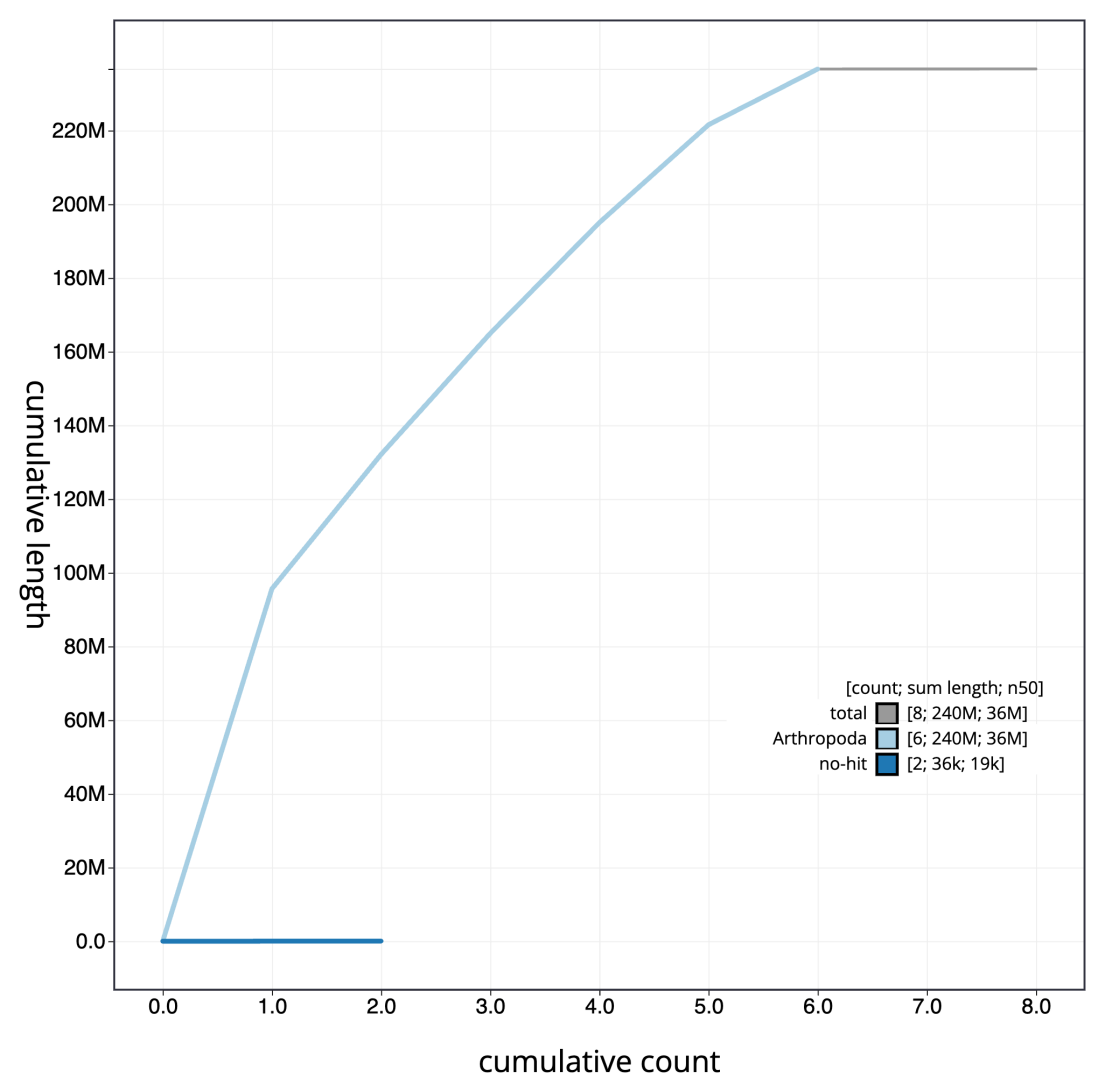
Genome assembly of *Malthinus flaveolus*, icMalFlav1.1: BlobToolKit cumulative sequence plot. The grey line shows cumulative length for all sequences. Coloured lines show cumulative lengths of sequences assigned to each phylum using the buscogenes taxrule. An interactive version of this figure is available at https://blobtoolkit.genomehubs.org/view/icMalFlav1_1/dataset/icMalFlav1_1/cumulative.

**Figure 5.**
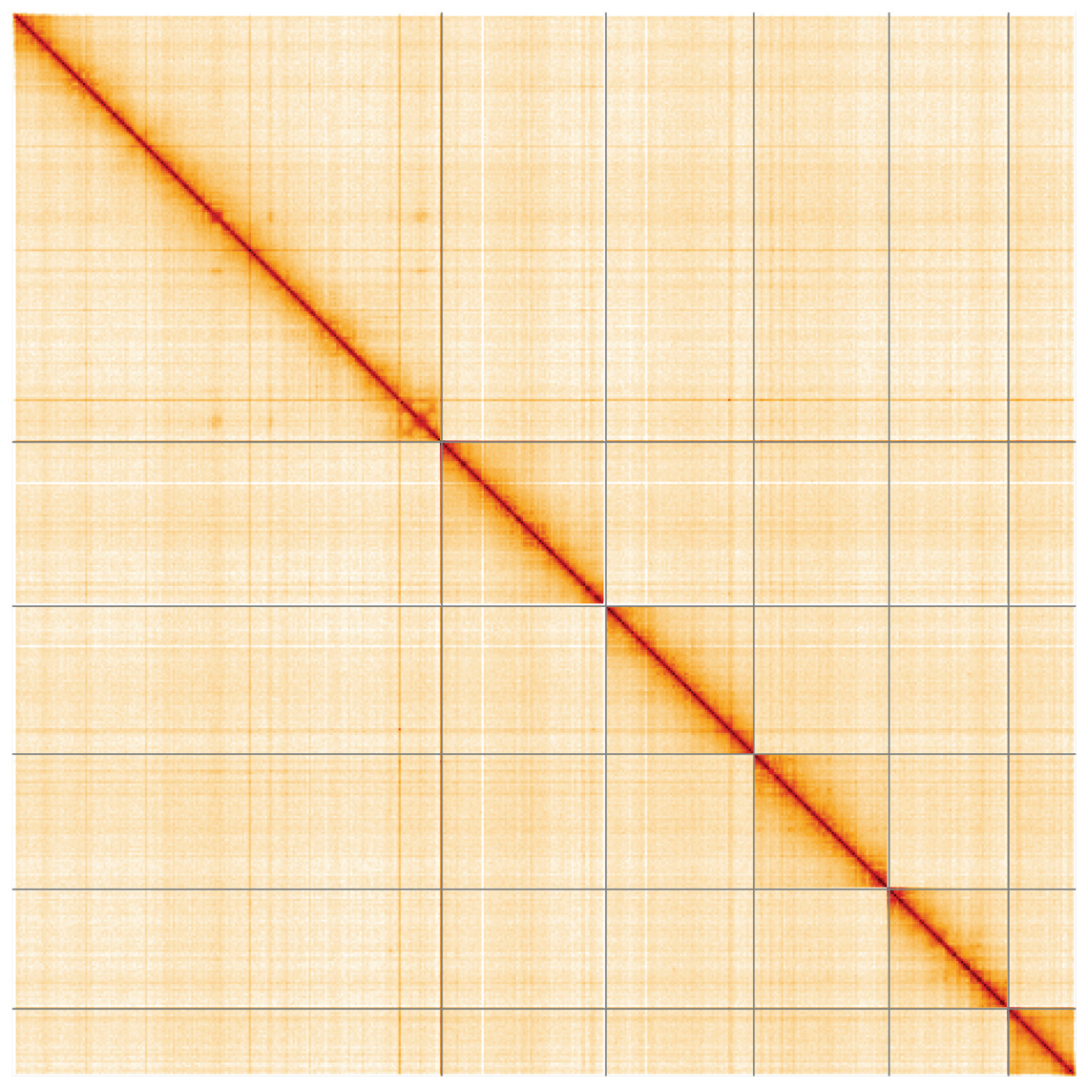
Genome assembly of *Malthinus flaveolus*, icMalFlav1.1: Hi-C contact map of the icMalFlav1.1 assembly, visualised using HiGlass. Chromosomes are shown in order of size from left to right and top to bottom. An interactive version of this figure may be viewed at https://genome-note-higlass.tol.sanger.ac.uk/l/?d=eujcxwmbQrixlQ_Cei5ejA.

The final assembly has a total length of 236.7 Mb in 7 sequence scaffolds with a scaffold N50 of 36.5 Mb ( Table 1). The snail plot in Figure 2 provides a summary of the assembly statistics, while the distribution of assembly scaffolds on GC proportion and coverage is shown in Figure 3. The cumulative assembly plot in Figure 4 shows curves for subsets of scaffolds assigned to different phyla. Most (99.98%) of the assembly sequence was assigned to 6 chromosomal-level scaffolds, representing 5 autosomes and the X sex chromosome. Chromosome-scale scaffolds confirmed by the Hi-C data are named in order of size ( Figure 5; Table 2). The X chromosome was identified based on synteny with the genome assembly of *Cantharis rufa* (GCA_947369205.1) ( Sivell *et al.*, 2023). While not fully phased, the assembly deposited is of one haplotype. Contigs corresponding to the second haplotype have also been deposited. The mitochondrial genome was also assembled and can be found as a contig within the multifasta file of the genome submission.

**Table 1.** Genome data for *Malthinus flaveolus*, icMalFlav1.1.

Project accession data		
Assembly identifier	icMalFlav1.1	
Species	*Malthinus flaveolus*	
Specimen	icMalFlav1	
NCBI taxonomy ID	195224	
BioProject	PRJEB60643	
BioSample ID	SAMEA10978905	
Isolate information	icMalFlav1, female: whole organism (DNA and Hi-C sequencing)	
Assembly metrics *		*Benchmark*
Consensus quality (QV)	66.8	*≥ 50*
*k*-mer completeness	100.0%	*≥ 95%*
BUSCO **	C:99.0%[S:98.1%,D:0.9%],F:0.5%,M:0.5%,n:2,124	*C ≥ 95%*
Percentage of assembly mapped to chromosomes	99.98%	*≥ 95%*
Sex chromosomes	X	*localised homologous pairs*
Organelles	Mitochondrial genome: 19.27 kb	*complete single alleles*
Raw data accessions		
PacificBiosciences SEQUEL II	ERR11029662	
Hi-C Illumina	ERR11040174	
Genome assembly		
Assembly accession	GCA_950108345.1	
*Accession of alternate haplotype*	GCA_950111595.1	
Span (Mb)	236.7	
Number of contigs	106	
Contig N50 length (Mb)	3.5	
Number of scaffolds	7	
Scaffold N50 length (Mb)	36.5	
Longest scaffold (Mb)	95.64	
Genome annotation		
Number of protein-coding genes	16,617	
Number of gene transcripts	16,934	

* Assembly metric benchmarks are adapted from column VGP-2020 of “Table 1: Proposed standards and metrics for defining genome assembly quality” from Rhie *et al.* (2021). ** BUSCO scores based on the endopterygota_odb10 BUSCO set using version 5.3.2. C = complete [S = single copy, D = duplicated], F = fragmented, M = missing, n = number of orthologues in comparison. A full set of BUSCO scores is available at https://blobtoolkit.genomehubs.org/view/icMalFlav1_1/dataset/icMalFlav1_1/busco.

**Table 2.** Chromosomal pseudomolecules in the genome assembly of *Malthinus flaveolus*, icMalFlav1.

INSDC accession	Chromosome	Length (Mb)	GC%
OX467285.1	1	95.64	34.0
OX467286.1	2	36.46	34.0
OX467287.1	3	32.85	34.0
OX467288.1	4	30.07	34.5
OX467289.1	5	26.55	35.0
OX467290.1	X	15.12	34.5
OX467291.1	MT	0.02	25.0

The estimated Quality Value (QV) of the final assembly is 66.8 with *k*-mer completeness of 100.0%, and the assembly has a BUSCO v5.3.2 completeness of 99.0% (single = 98.1%, duplicated = 0.9%), using the endopterygota_odb10 reference set ( *n* = 2,124).

Metadata for specimens, barcode results, spectra estimates, sequencing runs, contaminants and pre-curation assembly statistics are given at https://links.tol.sanger.ac.uk/species/195224.

## Genome annotation report

The *Malthinus flaveolus* genome assembly (GCA_950108345.1) was annotated at the European Bioinformatics Institute (EBI) using the Ensembl rapid annotation pipeline. The resulting annotation includes 16,934 transcribed mRNAs from 16,617 protein-coding genes ( Table 1; https://rapid.ensembl.org/Malthinus_flaveolus_GCA_950108345.1/Info/Index).

## Methods

### Sample acquisition and nucleic acid extraction

A female *Malthinus flaveolus* (specimen ID Ox001636, ToLID icMalFlav1) was collected from Wytham Woods, Oxfordshire (biological vice-county Berkshire), UK (latitude 51.77, longitude –1.33) on 2021-07-08 by potting. The specimen was collected and identified by Mark Telfer (independent researcher), and then snap-frozen on dry ice. 

The workflow for high molecular weight (HMW) DNA extraction at the Wellcome Sanger Institute (WSI) includes a sequence of core procedures: sample preparation; sample homogenisation, DNA extraction, fragmentation, and clean-up. In sample preparation, the icMalFlav1 sample was weighed and dissected on dry ice ( Jay *et al.*, 2023). Tissue from the whole organism was homogenised using a PowerMasher II tissue disruptor ( Denton *et al.*, 2023a).

HMW DNA was extracted in the WSI Scientific Operations core using the Automated MagAttract v2 protocol ( Oatley *et al.*, 2023). The DNA was sheared into an average fragment size of 12–20 kb in a Megaruptor 3 system with speed setting 31 ( Bates *et al.*, 2023). Sheared DNA was purified by solid-phase reversible immobilisation ( Strickland *et al.*, 2023): in brief, the method employs a 1.8X ratio of AMPure PB beads to sample to eliminate shorter fragments and concentrate the DNA. The concentration of the sheared and purified DNA was assessed using a Nanodrop spectrophotometer and Qubit Fluorometer and Qubit dsDNA High Sensitivity Assay kit. Fragment size distribution was evaluated by running the sample on the FemtoPulse system.

Protocols developed by the WSI Tree of Life laboratory are publicly available on protocols.io ( Denton *et al.*, 2023b).

### Sequencing

Pacific Biosciences HiFi circular consensus DNA sequencing libraries were constructed according to the manufacturers’ instructions. DNA sequencing was performed by the Scientific Operations core at the WSI on a Pacific Biosciences SEQUEL II instrument. Hi-C data were also generated from remaining tissue of icMalFlav1 using the Arima2 kit and sequenced on the Illumina NovaSeq 6000 instrument.

### Genome assembly, curation and evaluation

Assembly was carried out with Hifiasm ( Cheng *et al.*, 2021) and haplotypic duplication was identified and removed with purge_dups ( Guan *et al.*, 2020). The assembly was then scaffolded with Hi-C data ( Rao *et al.*, 2014) using YaHS ( Zhou *et al.*, 2023). The assembly was checked for contamination and corrected as described previously ( Howe *et al.*, 2021). Manual curation was performed using HiGlass ( Kerpedjiev *et al.*, 2018) and PretextView ( Harry, 2022). The mitochondrial genome was assembled using MitoHiFi ( Uliano-Silva *et al.*, 2023), which runs MitoFinder ( Allio *et al.*, 2020) or MITOS ( Bernt *et al.*, 2013) and uses these annotations to select the final mitochondrial contig and to ensure the general quality of the sequence.

A Hi-C map for the final assembly was produced using bwa-mem2 ( Vasimuddin *et al.*, 2019) in the Cooler file format ( Abdennur & Mirny, 2020). To assess the assembly metrics, the *k*-mer completeness and QV consensus quality values were calculated in Merqury ( Rhie *et al.*, 2020). This work was done using Nextflow ( Di Tommaso *et al.*, 2017) DSL2 pipelines “sanger-tol/readmapping” ( Surana *et al.*, 2023a) and “sanger-tol/genomenote” ( Surana *et al.*, 2023b). The genome was analysed within the BlobToolKit environment ( Challis *et al.*, 2020) and BUSCO scores ( Manni *et al.*, 2021; Simão *et al.*, 2015) were calculated.

Table 3 contains a list of relevant software tool versions and sources.

**Table 3.** Software tools: versions and sources.

Software tool	Version	Source
BlobToolKit	4.2.1	https://github.com/blobtoolkit/blobtoolkit
BUSCO	5.3.2	https://gitlab.com/ezlab/busco
Hifiasm	0.16.1-r375	https://github.com/chhylp123/hifiasm
HiGlass	1.11.6	https://github.com/higlass/higlass
Merqury	MerquryFK	https://github.com/thegenemyers/MERQURY.FK
MitoHiFi	3	https://github.com/marcelauliano/MitoHiFi
PretextView	0.2	https://github.com/wtsi-hpag/PretextView
purge_dups	1.2.5	https://github.com/dfguan/purge_dups
sanger-tol/genomenote	v1.0	https://github.com/sanger-tol/genomenote
sanger-tol/readmapping	1.1.0	https://github.com/sanger-tol/readmapping/tree/1.1.0
YaHS	1.2a	https://github.com/c-zhou/yahs

### Genome annotation

The BRAKER2 pipeline ( Brůna *et al.*, 2021) was used in the default protein mode to generate annotation for the *Malthinus flaveolus* assembly (GCA_950108345.1) in Ensembl Rapid Release at the EBI.

### Wellcome Sanger Institute – Legal and Governance

The materials that have contributed to this genome note have been supplied by a Darwin Tree of Life Partner. The submission of materials by a Darwin Tree of Life Partner is subject to the **‘Darwin Tree of Life Project Sampling Code of Practice’**, which can be found in full on the Darwin Tree of Life website here. By agreeing with and signing up to the Sampling Code of Practice, the Darwin Tree of Life Partner agrees they will meet the legal and ethical requirements and standards set out within this document in respect of all samples acquired for, and supplied to, the Darwin Tree of Life Project. 

Further, the Wellcome Sanger Institute employs a process whereby due diligence is carried out proportionate to the nature of the materials themselves, and the circumstances under which they have been/are to be collected and provided for use. The purpose of this is to address and mitigate any potential legal and/or ethical implications of receipt and use of the materials as part of the research project, and to ensure that in doing so we align with best practice wherever possible. The overarching areas of consideration are:

•    Ethical review of provenance and sourcing of the material

•    Legality of collection, transfer and use (national and international) 

Each transfer of samples is further undertaken according to a Research Collaboration Agreement or Material Transfer Agreement entered into by the Darwin Tree of Life Partner, Genome Research Limited (operating as the Wellcome Sanger Institute), and in some circumstances other Darwin Tree of Life collaborators.

## Data Availability

European Nucleotide Archive: *Malthinus flaveolus*. Accession number PRJEB60643; https://identifiers.org/ena.embl/PRJEB60643 ( Wellcome Sanger Institute, 2023). The genome sequence is released openly for reuse. The *Malthinus flaveolus* genome sequencing initiative is part of the Darwin Tree of Life (DToL) project. All raw sequence data and the assembly have been deposited in INSDC databases. Raw data and assembly accession identifiers are reported in Table 1.

## Author information

Members of the University of Oxford and Wytham Woods Genome Acquisition Lab are listed here: https://doi.org/10.5281/zenodo.7125292.

Members of the Darwin Tree of Life Barcoding collective are listed here: https://doi.org/10.5281/zenodo.4893703.

Members of the Wellcome Sanger Institute Tree of Life Management, Samples and Laboratory team are listed here: https://doi.org/10.5281/zenodo.10066175.

Members of Wellcome Sanger Institute Scientific Operations: Sequencing Operations are listed here: https://doi.org/10.5281/zenodo.10043364.

Members of the Wellcome Sanger Institute Tree of Life Core Informatics team are listed here: https://doi.org/10.5281/zenodo.10066637.

Members of the Tree of Life Core Informatics collective are listed here: https://doi.org/10.5281/zenodo.5013541.

Members of the Darwin Tree of Life Consortium are listed here: https://doi.org/10.5281/zenodo.4783558.
