# Supplementary material for: The genome sequence of a soldier beetle, Malthinus flaveolus (Herbst, 1786)
Source: Wellcome Open Res. 2024 Mar 1;9:121. [Version 1] doi: 10.12688/wellcomeopenres.21086.1 (PMC11411255; doi:10.12688/wellcomeopenres.21086.1)
Supplement: Peer review history

# Reviewer report: Reviewer response for version 1

**DOI:** 10.21956/wellcomeopenres.23326.r96343

**Published:** 2024-09-20

**Authors:** Jose Manuel Rico Porras

**Affiliations:** 1 Universidad de Jaen, Jaén, Andalusia, Spain

The article accurately describes the steps used for the assembly of the genome of the soldier beetle *Malthinus flaveolus*. Some points to consider:

- Include the family and order in the title: *The genome sequence of a soldier beetle, Malthinus flaveolus (Herbst, 1786) (Coleoptera, Cantharidae)*
- Mention in the Background section that there are no cytogenetic data available for this species (Coleoptera Karyotype Database ^1^), but data are available for the species *Malthinus seriepunctatus ^2^*.

Are sufficient details of methods and materials provided to allow replication by others?

Yes

Is the rationale for creating the dataset(s) clearly described?

Yes

Are the datasets clearly presented in a useable and accessible format?

Yes

Are the protocols appropriate and is the work technically sound?

Yes

Reviewer Expertise:

Cytogenomics, repetitive DNA

I confirm that I have read this submission and believe that I have an appropriate level of expertise to confirm that it is of an acceptable scientific standard.

## References

[1] : Coleoptera Karyotype Database. *The Coleopterists Bulletin* .2015;69(1) : 10.1649/0010-065X-69.1.174 174-175 10.1649/0010-065X-69.1.174

[2] : A chromosomal investigation of some British Cantharidae (Coleoptera). *Genetica* .2007;130(3) : 10.1007/s10709-006-9106-5 293-300 10.1007/s10709-006-9106-5 17004007

# Reviewer report: Reviewer response for version 1

**DOI:** 10.21956/wellcomeopenres.23326.r94359

**Published:** 2024-09-18

**Authors:** Yangzi Wang

**Affiliations:** 1 Johannes Gutenberg University Mainz Institute of Organismic and Molecular Evolution (Ringgold ID: 163392), Mainz, Rhineland-Palatinate, Germany

The article presents a high-quality genome assembly of *Malthinus flaveolus*, generated using Pacific Biosciences HiFi long-read sequencing and Hi-C chromosomal conformation capture. This work has resulted in a valuable genomic resource for the scientific community, providing the first complete genome assembly and gene annotation for this species. The raw sequencing data, the final assembled genome, and the gene annotations are well documented and deposited in public database, making the data easily accessible for future research. Overall, the project appears to have been conducted with a high level of professionalism and technical expertise.

 However, there are some issues in the manuscript that prevent it from being suitable for Indexing at this stage:

**1. Typos, e.g.:**

 In the first paragraph of “Background”, the authors mention three subfamilies within the family Cantharidae: "Cantharidae, with 24 species, Silinae with just one (Silis ruficollis), and the remaining 16 species are classified under Malthininae." Here, "Cantharidae" seems incorrect. Please review and correct this.

 Third Paragraph of “Background”: The sentence "A key feature of the British Malthininae are the somewhat shortened elytra, which leave a part of the hind wings exposed when the beetle is resting." should use "is" instead of "are".

**2. An issue concerning the identification of sex chromosome:**

 In the "genome sequence report" section, the manuscript states, "The X chromosome was identified based on synteny with the genome assembly of Cantharis rufa (GCA_947369205.1) (Sivell et al., 2023)." While it is exciting that the authors have attempted to identify the sex chromosome, the reference paper for the Cantharis rufa genome (Sivell et al., 2023) does not provide a clear explanation of how the sex chromosomes were identified. This makes the current claim less reliable. The authors should provide more robust evidence or additional analyses to support the identification of the sex chromosome.

Are sufficient details of methods and materials provided to allow replication by others?

Yes

Is the rationale for creating the dataset(s) clearly described?

Yes

Are the datasets clearly presented in a useable and accessible format?

Yes

Are the protocols appropriate and is the work technically sound?

Partly

Reviewer Expertise:

genomics, population genomics, epigenomics

I confirm that I have read this submission and believe that I have an appropriate level of expertise to confirm that it is of an acceptable scientific standard.

# Reviewer report: Reviewer response for version 1

**DOI:** 10.21956/wellcomeopenres.23326.r96342

**Published:** 2024-09-16

**Authors:** Na Ra Shin

**Affiliations:** 1 University of Memphis, Memphis, USA

This data note provides a high-quality reference genome assembly for the soldier beetle, *Malthinus flaveolus*, and includes a detailed introduction to the species. The clarity of the data note is enhanced by its comprehensive description of sample information, HMW DNA extraction procedures, and sequencing steps.

**Minor comments:**

 1) It would be helpful to include the BUSCO score in the abstract.

 2) Please ensure that the full name is provided before using any abbreviations.

 (e.g., *M. punctatus*)

Are sufficient details of methods and materials provided to allow replication by others?

Yes

Is the rationale for creating the dataset(s) clearly described?

Yes

Are the datasets clearly presented in a useable and accessible format?

Yes

Are the protocols appropriate and is the work technically sound?

Yes

Reviewer Expertise:

Insect genome and transcriptome

I confirm that I have read this submission and believe that I have an appropriate level of expertise to confirm that it is of an acceptable scientific standard.
